# The practice of universal screening for Lynch syndrome in newly diagnosed endometrial carcinoma

**DOI:** 10.1002/hsr2.43

**Published:** 2018-06-14

**Authors:** Aifen Wang, Jenna McCracken, Yanjing Li, Lingfan Xu

**Affiliations:** ^1^ Department of Obstetrics and Gynecology, Zhangjiagang Hospital Nanjing University of Chinese Medicine Jiangsu China 215600; ^2^ Department of Pathology Duke University School of Medicine Durham NC USA

**Keywords:** endometrial cancer, hereditary nonpolyposis cancer syndrome, Lynch syndrome, microsatellite instability, mismatch repair proteins

## Abstract

**Background:**

Lynch syndrome (LS) accounts for 5% of all endometrial cancer (EC) cases and 4% of all lifetime risk of developing colorectal cancer. While current guidelines recommend LS screening for all patients with newly diagnosed colorectal cancer, there is no such guideline for screening patients with EC.

**Discussion:**

This review addresses LS screening and discusses algorithms for testing patients in the setting of newly diagnosed EC.

**Conclusion:**

The successful diagnosis of LS has important implications, including prevention of LS‐associated cancers among relatives and immunotherapy recommendations for patients with advanced EC and loss of expression of mismatch repair immunohistochemistry and microsatellite instability positive following failure of traditional treatment.

## 1 INTRODUCTION

Endometrial cancer (EC) is the third most common malignancy in women worldwide. It is also the most common gynecologic malignancy in developed countries and the second most common gynecologic malignancy in the developing world. In the United States, there are over 60 000 new diagnoses and over 10 000 deaths from this disease each year.^1^ One of the underlying causes of EC is mutation of DNA mismatch repair (MMR) genes. Lynch syndrome (LS), also known as Hereditary Nonpolyposis Colorectal Carcinoma, is caused by germline mutations in the same MMR genes. Notably, female relatives inheriting the germline mutation have a high risk of developing endometrial carcinoma, about 43% by age 75.^2^

Formally established as a recommendation in 2009, patients with newly diagnosed colorectal carcinoma are now universally screened for MMR mutations, using either the Amsterdam or the Bethesda criteria.^3^ Multiple factors are used to identify patients who would benefit from screening, including personal history, age, family history, and histological morphology.^4^, ^5^, ^6^ The Society of Gynecologic Oncologists has established guidelines for screening patients with gynecologic tumors (Table 1)^7^; however, these may miss the diagnosis of LS in patients with EC because of the narrow screening criteria.

**Table 1:** Society of Gynecologic Oncology (SGO) guidelines for referral for Lynch syndrome counseling^a^

● Patients with endometrial or colorectal cancer with evidence of microsatellite instability or loss of DNA mismatch repair protein expression (MLH1, MSH2, MSH6, PMS2) on immunohistochemistry
● Patients with a first‐ or second‐degree relative with a known mutation in a mismatch repair gene.
● Families with few female relatives, as this may lead to an underrepresentation of female cancers despite the presence of a predisposing family mutation.
● Hysterectomy and/or oophorectomy at a young age in multiple family members, as this might mask a hereditary gynecologic cancer predisposition.
● Presence of adoption in the lineage.

a Patients with an increased likelihood of Lynch syndrome and for whom genetic assessment is recommended. Data from Lancaster JM, Powell CB, Chen LM, et al. Society of Gynecologic Oncology statement on risk assessment for inherited gynecologic cancer predispositions. Gynecol Oncol 2015;136:3–7.

Studies examining the prevalence of MMR mutations among EC tumors show that it ranges between 2% and 5.9%.^8^, ^9^, ^10^, ^11^, ^12^, ^13^, ^14^ When patients with ovarian cancer are also included, a study of over 580 patients at the University of Hospital of Dresden, by Kast et al, estimated the rate of pathogenic germline mutations to be of only 0.3%.^15^ A study conducted by Dillon et al^14^ showed that 2.1% (5/233) of EC patients were identified to have LS, with germline MMR mutations; 1.3% (3/233) patients were found to have Lynch‐like syndrome,^14^ negative for germline MMR mutations. One patient with Lynch‐like syndrome was identified to have biallelic somatic mutation.

In patients with LS, colorectal and endometrial cancers are the most common “sentinel cancers” with which patients present to clinical attention.^7^, ^11^, ^16^, ^17^, ^18^, ^19^ The most common and clinically relevant mutations in LS occur in the *MLH1*, *MSH2*, *MSH6*, *PMS2*, and *EPCAM* genes.^20^, ^21^ A report from the prospective LS database followed over 3000 patients for a total of 24 475 person years and determined the cumulative incidences of EC (at age 75) to be 43%, 57%, and 46%, for patients with mutations in *MLH1*, *MSH2*, and *MSH6*, respectively.^2^

There are significant clinical advantages to detecting LS among patients with newly diagnosed EC. First, surveillance testing can be performed for colorectal carcinoma and other LS‐associated cancers. Second, relatives have the opportunity for genetic counseling, surveillance testing, and even risk‐reducing surgeries to prevent LS‐associated cancers. Third, patients dually diagnosed with endometrial and colorectal carcinoma can receive optimized treatment for colorectal cancer. Lastly, PD‐1/PD‐L1 checkpoint blockade may be a good choice for the treatment of EC in patients with defective MMR or microsatellite instability (MSI), following failure of traditional treatment.^12^, ^22^, ^23^, ^24^, ^25^

## 2 CLINICOPATHOLOGIC FEATURES OF ENDOMETRIAL CARCINOMA IN THE CONTEXT OF LYNCH SYNDROME

Multiple studies have identified that the lower uterine segment is more frequently involved in endometrial carcinoma in patients with MMR mutations than in the general population with EC. Westin et al determined that the lower uterine segment is affected in 29% of patients with LS, yet only in 1.8% of the general EC population.^26^, ^27^ Interestingly, Masuda et al^28^ identified lower uterine segment involvement in only 11.1% of Asian LS patients. Two studies have shown lower uterine segment involvement in only a small fraction of cases of endometrial carcinoma with *MLH1* methylation.^29^, ^30^ There is a wide spectrum of histopathology among ECs with MMR germline mutations, including endometrioid, clear cell, serous, mixed (endometrioid and clear cell) carcinomas, and carcinosarcomas.^30^ Karamurzin et al^31^ conducted a study of 25 patients with defective MMR who underwent prophylactic hysterectomy and bilateral salpingo‐oophorectomy at Memorial Sloan‐Kettering Cancer Center, and identified 2 cases of focal FIGO grade 1 endometrioid EC, 3 cases of focal complex atypical hyperplasia, and 1 case of endometrioid/clear cell ovarian cancer.

Endometrial cancers with MMR mutations have several distinctive histological features, including prominent peritumoral lymphocytes, tumor‐infiltrating lymphocytes, tumor heterogeneity, and undifferentiated/dedifferentiated morphologies.^32^ Endometrial cancer tumors with MMR gene mutations are also more likely to exhibit MSI.^32^

## 3 TESTING FOR LYNCH SYNDROME

In developing a cost‐effective screening strategy, one must consider both the proper patient populations to include and exclude, as well as the optimal testing technology. Studies have repeatedly shown that the existing Amsterdam and Bethesda criteria fail to identify a substantial number of LS patients.^27^, ^33^, ^34^ Although these criteria generally select for patients under the age of 50, a recent study of 1002 patients with EC by Goodfellow et al^35^ determined that 24% of patients with MMR germline mutations are older than 60 years old. A second study of patients with germline MMR gene mutations, by Mills et al,^23^ revealed that 75% of affected women were older than 50 years old and, furthermore, that 85% of women had no history of previous malignancy. Critically, approximately 40% patients with LS‐associated germline MMR mutations lacked the traditional indicators recommended for LS screening, such as age under 50, typical tumor histology, lower uterine segment involvement, and a positive cancer pedigree (as defined by the Bethesda criteria).^27^ Ring et al randomly screened 381 patients with newly diagnosed EC for MMR protein deficiencies, methylation, and mutation testing and identified 22 (5.8%) with LS. Eight patients were older than 50 years, and 10 patients had no family history of LS‐associated malignancy.^33^ These findings emphasize the considerable number of patients whose diagnosis of LS would be missed by the existing criteria.

Germline mutation sequencing is the gold standard test for confirming the diagnosis of LS but is currently too expensive to be used as a screening tool. The sensitivity and specificity of MSI testing vary depending on the specific mutated gene: While testing is 80% to 91% sensitive and 90% specific for *MLH1* or *MSH2* mutations, it is 55% to 77% sensitive and 90% specific for *MSH6* or *PMS2* mutations.^3^ By contrast, immunohistochemistry (IHC) testing is 83% sensitive and 89% specific, regardless of which MMR gene is involved.^3^ In addition to its applicability to all mutated genes, IHC is also faster to perform, less expensive, and easier to test on biopsy samples than MSI.^36^

The aforementioned study by Goodfellow et al tested a combination of techniques, including MMR protein IHC, *MLH1* methylation analysis, and MSI testing.^35^ The study identified a patient who initially tested negative for LS using the IHC method, but positive via MSI, highlighting the differences in sensitivity among assays. A separate study analyzed blood samples from patients with EC for pathogenic MMR mutations via sequencing and compared them with tumor tissue‐based MSI and IHC analysis. Among those women with MMR mutations, only 41.66% of samples were positive via MSI and 76.92% positive via IHC,^34^ further underscoring the need to test EC patients using a combination of techniques.

In light of the number of LS patients that are excluded from testing under the Amsterdam and Bethesda clinical criteria and the limitations of a single‐test model, multiple studies have investigated the feasibility of laboratory‐based screening of all endometrial cancer patients using a multitest screening approach of all endometrial cancer patients. Buchanan et al^37^ determined that testing with a combination of IHC and *MLH1* methylation yielded the higher positive predictive value to identify MMR mutation carriers compared with MSI by polymerase chain reaction (PCR) testing. The Bethesda panel is a PCR‐based approach to test *MSI* at 5 microsatellites loci, consisting of 3 dinucleotide and 2 mononucleotide repeats, aimed at determining differences in repeat number between tumor and nontumor regions.^38^ Dinucleotide repeats have been reported to have less sensitivity and specificity for identifying MSI than mononucleotide repeats,^38^ particularly in patients with underlying *MSH6* mutations.^38^, ^39^, ^40^, ^41^ The pentaplex panel uses 5 mononucleotide repeats as markers.^38^, ^39^, ^40^, ^41^ It shows higher sensitivity and specificity than the Bethesda panel and has been recommended instead of the Bethesda panel.^38^, ^39^, ^40^, ^41^ An hexaplex panel, developed by Pagin et al, uses 6 mononucleotide repeats as markers and shows higher sensitivity and specificity than the pentaplex panel in patients with *MSH6* and noncolon cancer.^42^ The MOSAIC method developed by Hause et al has a high sensitivity and specificity in identifying MSI‐H tumors, especially in endometrial cancer among 18 types of tumors.^43^

**Figure 1:**
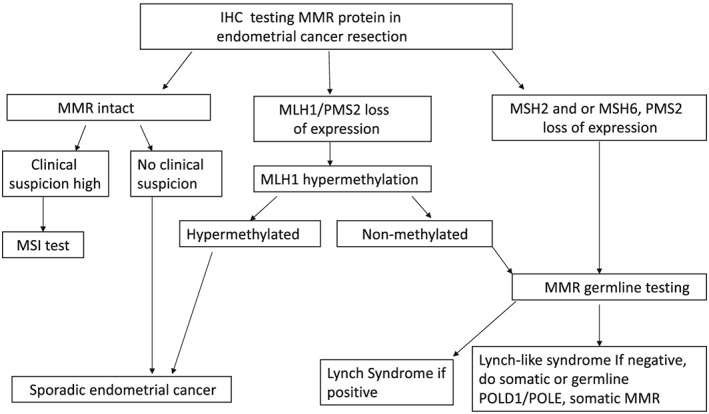
Algorithm for universal Lynch syndrome screening in newly diagnosed endometrial cancer

Here, we suggest following the screening strategy outlined in Figure 1. First, use MMR IHC to identify patients who have lost expression of *MLH1*, *MSH2*, *PMS2*, or *MSH6*. Second, those with loss of *MLH1* and *PMS2* expression should subsequently be tested for *MLH1* methylation. Those with intact expression of MMR but with high clinical suspicion for LS should undergo MSI testing. Third, the resultant subset of patients for whom *MLH1* methylation is negative should then receive confirmatory MMR mutation sequencing. By contrast, patients with loss of *MSH2* and/or *MSH6* expression via IHC should proceed directly to germline mutation sequencing. Lastly, in cases of negative germline testing for *MLH1*, *MSH2*, *MSH6*, and *PMS2*, testing should be expanded to include sequencing of the exonuclease domains (EDMs) of DNA polymerases Pol ɛ (*POLE*) and δ (*POLD1*).

There are 3 possible outcomes of the above testing algorithm: LS, Lynch‐like syndrome—also known as suspected LS (sLS)—and sporadic EC. Lynch‐like syndrome is nearly identical to LS, as both tumor types have loss of MMR gene expression (via IHC) and are *MHL1* methylation negative, but Lynch‐like syndrome patients do not have germline MMR mutations.^44^, ^45^, ^46^ Jansen et al^47^ analyzed leukocyte and tumor DNA of 62 sLS patients using gene panel sequencing including the *POLE*, *POLD1*, and MMR genes. Sixty‐four percent of tumors showed either 1, 2, or more somatic MMR variants predicted to affect function. Fourteen percent sLS tumors showed a likely ultramutated phenotype and were found to carry germline (n = 2) or somatic variants (n = 7) in the *POLE/POLD1* EDM. Sixty‐seven percent of these *POLE/POLD1‐EDM* mutated tumors also carried somatic MMR variants. This finding suggested that faulty proofreading may result in loss of MMR and, thereby, in MSI. Haraldsdottir et al^48^ found that almost 70% of patients with Lynch‐like syndrome acquire somatic mutations in MMR genes, leading to an hypermutated phenotype.

It is believed that Lynch‐like syndrome accounts for up to 71% of clinically suspected (but untested) LS patients.^44^ An Asian study showed that 4.7% patients were identified to have Lynch‐like syndrome among 360 women with EC.^49^ There are no significant clinicopathologic differences between Lynch‐like syndrome and LS, except that Lynch‐like syndrome patients are less likely to have LS‐associated cancers.^50^ Sporadic EC (also known as epigenetic endometrial carcinoma) is composed of 2 subtypes: one is IHC with loss of MMR protein expression and the other is IHC with intact *MLH1* and *PMS2* expression and positive testing for *MLH1* methylation. The second group is hypothesized to be related to biallelic *MSH2* somatic mutations.^51^ Endometrial cancer patients with sporadic mutations in MMR proteins typically present at a more advanced stage, with larger, higher grade tumors, and more lymph nodes involvement. These patients have an overall poorer prognosis and shorter recurrence‐free survival.^52^

Although seemingly counterintuitive, patients with LS may have multiple malignancies with different MMR gene signatures, thus necessitating rescreening and retesting with each subsequent tumor. In a study by Roth et al, of patients with synchronous or metachronus malignancies, 69% of patients showed concordant MMR results, whereas 31% showed discordant results.^53^ Thus, even if the first tumor has common variants of the wide‐type allele of the MMR genes, it is not uncommon for subsequent tumors to develop MMR mutations in the context of LS.

## 4 TREATMENT OF ENDOMETRIAL CARCINOMA WITH MUTATED MMR

Mismatch repair gene mutations and *MSI* are associated with unique responses to treatment. For example, the traditional treatment for complex atypical hyperplasia or well‐differentiated EC, progesterone, is not effective in patients younger than 55 years old with MMR mutations.^54^ Immune checkpoint inhibitors, like programmed cell death ligand‐1 (PD‐L1) inhibitor, are attractive therapeutic options for the treatment of advanced LS‐associated cancers, including colorectal cancer, prostate cancer, and melanoma, because they have fewer side effects than traditional chemotherapy. Indeed, patients with LS may be excellent candidates for PD‐L1 inhibitor therapy, as PD‐L1 is expressed in 52.6% of ECs with MMR mutations, as compared with 10% of those with normal MMR.^55^ In a separate study, endometrial and ovarian cancers with MMR mutations were associated with a more favorable prognosis.^56^ PD‐L1 expression, assessed via IHC, has been suggested as a promising biomarker for patient response to PD‐1/PD‐L1 checkpoint blockade immunotherapy; however, because of a number of issues, PD‐L1 staining may not be suitable for routine and accurate selection of patients.^57^ Microsatellite instability is a key predictor of immunotherapy response rate in 40% of colorectal carcinomas and 71% of noncolorectal carcinomas with MMR mutations.^58^ A study conducted by Howitt et al^59^ showed that *POLE* and MSI are associated with high neoantigen loads and number of tumor‐infiltrating lymphocytes, which is counterbalanced by overexpression of PD‐1 and PD‐L1. *POLE* and MSI tumors may be excellent candidates for PD‐1‐targeted immunotherapies. Importantly, these data provide a rationale for treating patients with advanced carcinoma, who would otherwise live only a few months, with immune checkpoint inhibitors despite failure of conventional therapy.

Similarly, research by Shikama et al demonstrated that patients with MMR protein deficiency had more favorable survival outcomes than did patients with sporadic EC.^60^ Germline mutations in *MSH2*, *MSH6*, *PMS2*, or *MHL1* (and absent *MHL1* methylation) were associated with younger age of onset, superficial tumor invasion, and early‐stage disease, whereas sporadic endometrial cancer was the opposite. Although these mutations also predisposed patients to having other LS‐associated malignancies, they were also more sensitive to adjuvant immune therapy than sporadic EC (with nonmutated MMR genes), resulting in an overall more favorable prognosis.

## 5 CANCER SURVEILLANCE IN PATIENTS WITH LYNCH SYNDROME

A multicenter study by Møller et al assessed cancer detection among LS patients undergoing prospective routine colonoscopic and gynecologic surveillance.^61^ The cohort included 1942 women who carried MMR gene mutations, without prior malignancies, observed for a total of 13 782 person years. A total of 314 patients developed cancer, mostly colorectal cancer (N = 151, 48%), endometrial cancer (N = 72, 22.9%), and ovarian cancer (N = 19, 6%). When the data were stratified according to specific gene mutations, alterations in *MLH1*, *MSH2*, *MSH6*, and *PMS2* resulted in a cumulative prevalence at age 70 of 34%, 51%, 49%, and 24%, respectively, for endometrial cancer, and 11%, 15%, 0%, 0%, respectively, for ovarian cancer. The overall 10‐year survival rate following the development of any cancer was 87%. However, if the sentinel cancer was colorectal, the 10‐year survival rate was extremely low (9%), while it was significantly higher for those who first developed endometrial cancer (98%) or ovarian cancer (89%).^56^

To monitor EC in patients with LS, we recommend the use of endometrial biopsy (with or without hysteroscopy), beginning at the age of 30 to 35 years, or 3 to 5 years prior to the age of onset of their youngest affected family member in premenopausal women, and traditional ultrasound, transvaginal ultrasound, and endometrial biopsy (with or without hysteroscopy) in postmenopausal women. The appropriate age at which to begin surveillance may vary for different MMR mutations. For example, *MSH6* mutation carriers could begin screening later than other MMR carriers.^56^ Similarly, those with truncating *MLH1* mutations could start surveillance later than those with nontruncating mutations.^62^

There is no consensus recommendation for decreasing the risk of endometrial carcinoma in women with LS, except for prophylactic hysterectomy. Recent investigations, however, indicate that hormone regulation and diabetes prevention may help to decrease risk.^63^ In 2015, a meta‐analysis showed that the duration of oral contraceptive use is proportional to the reduction in risk of endometrial cancer. It is estimated that, over the past decade, the increasing use of oral contraceptives in developed countries has protected approximately 400 000 women from EC.^64^ Although these benefits are not limited to women with MMR mutations, small studies have been conducted to specifically assess the potential benefits in the context of LS. A multicenter study by Lu et al^65^ compared levonorgestrel oral contraceptive pills with depo‐medroxyprogesterone acetate as chemoprevention for women with LS. They demonstrated that short‐term treatment with either oral contraceptive pills or depo‐medroxyprogesterone acetate reduced the proliferative response of the endometrium, indicating that progesterone may be an approach to prevent endometrial carcinoma in women with LS. A recent cohort study examined the associations between lifestyle, hormonal, reproductive, and medical factors, and the risk of EC in patients with LS.^66^ The results confirmed the association between EC and hormones, as women taking hormone replacement therapy were significantly more likely to develop EC. The same study also identified an association between type II diabetes and increased risk of EC. Thus, changes in lifestyle and medication are potential targets to reduce the risk of EC for patients with LS.

Prophylactic hysterectomy is an effective method of preventing EC. A retrospective cohort analysis examining the efficacy of risk‐reducing surgery identified 315 women with germline MMR mutations, of whom 61 underwent prophylactic hysterectomy, 46 had prophylactic bilateral salpingo‐oophorectomy, and 210 declined both procedures. Over the course of 3 decades, 33% of women who declined risk‐reducing surgery were diagnosed with EC. By contrast, 0% of women who underwent prophylactic surgery developed any gynecologic malignancy.^67^ Interestingly, a study by Bartosch et al^68^ discovered that among women with LS who underwent prophylactic hysterectomy, at least 23% already had abnormal pathologic endometrial findings of which they were clinically unaware, including EC, atypical hyperplasia, and nonatypical hyperplasia. Similar research by Downes et al detected endometrial cancer or precursor lesions in 32% of prophylactic hysterectomy specimens from women with LS.^69^ Unlike patients with *BRCA1* and *BRCA2* mutations, there are no formal recommendations regarding risk‐reducing gynecologic surgeries for patients with MMR mutations, despite evidence of benefit. A study of LS patients in the United Kingdom discovered that women who underwent prophylactic hysterectomy or salpingo‐oophorectomy felt significant emotional relief with respect to cancer risk reduction; however, menopausal symptoms and negative effects on body image were felt to adversely affect their quality of life.^70^ Although prophylactic hysterectomy is well documented as a successful way to reduce the risk of EC among LS patients, health professionals should continue to counsel patients regarding potential side effects and hormone replacement therapy.

## 6 SUMMARY

Endometrial cancer is the most common gynecological cancer in developed countries, of which 2% to 5.9% of cases are attributable to LS; patients with LS have a 40% to 60% lifetime risk of developing EC. In the context of LS, EC may display a wide range of histologic morphologies and is most tightly associated with mutations in *MSH2* and *MSH6*. In the clinic, we may use MMR IHC, *MLH1* promotor hypermethylation, MMR germline testing, *MSI*, somatic and or germline *POLD1* and *POLE* screening, and somatic MMR testing in all patients with newly diagnosed EC to identify sporadic endometrial cancer, LS, or Lynch‐like syndrome. We suggest women with LS who no longer desire childbearing to undergo risk‐reducing surgeries including hysterectomy and salpingo‐oophorectomy. Those who opt not to have prophylactic procedures should have regular cancer surveillance using ultrasound, transvaginal ultrasound, or endometrial biopsy (with or without hysteroscopy). PD‐1/PD‐L1 checkpoint blockade may be a good choice for the treatment in EC with MSI and loss of MMR expression, following failure of traditional treatment.

## CONFLICT OF INTEREST

None declared.

## AUTHOR CONTRIBUTIONS

Conceptualization: Aifen Wang

Writing—review and editing: Aifen Wang, Jenna McCracken, Yanjing Li, Lingfan Xu

Writing—original draft: Aifen Wang
